# Deaths in Buffalo in the Month of May

**Published:** 1865-06

**Authors:** 


### *Deaths in Buffalo in the month of May.*

Causes of Death.—Accident by drowning 8, anæmia 1, apoplexy, cerebral 1, brain, congestion of 3, brain, softening of 1, cancer of the stomach 1, cancer of the breast 1, cholera infantum 2, cholera morbus 1, consumption 13, convulsions 7, croup 3, croup, diphtheritic 1, debility 2, delirium tremens 1, diarrhœa 6, disease of the heart 4, disease of the stomach 1, diphtheria 4, dropsy, general 2, dropsy abdominal 1, dropsy of the heart 1, dysentery 1, fever 1, fever, puerperal 1, fever, scarlet 3, fever, typhoid 5, hemorrhage 1, inflammation of bowels 1, do. brain and meninges 5, do. liver 1, do. lungs 15, do. lungs, typhoid 3, do. lungs and pleura 1, inanition 1, kidneys, Bright's disease of 1, meningitis, cere. spin. 1, marasmus 1, measles 2, old age 3, premature birth 1, pyæmia 1, stomatitis 1, small-pox 10, suicide 1, syncope 1, tabes mesenterica 1, unknown 4, whooping cough 2. Total 136.

By whom Certified.—By regular physicians at public institutions 20; do. in city at large 63; by irregular practitioners 24; by coroner 11; by undertakers 18.

